# Patient engagement in clinical trial design for rare neuromuscular disorders: impact on the DELIVER and ACHIEVE clinical trials

**DOI:** 10.1186/s40900-023-00535-1

**Published:** 2024-01-02

**Authors:** Patricia Furlong, Ashish Dugar, Molly White

**Affiliations:** 1 Parent Project Muscular Dystrophy, 1012 14th NW, Suite 500, Washington, DC 20005 USA; 2 Dyne Therapeutics, Inc., Waltham, MA USA

**Keywords:** Patient-focused drug development, Patient and public involvement, Duchenne muscular dystrophy, Myotonic dystrophy

## Abstract

**Background:**

Engaging individuals living with disease in drug development and regulatory processes leads to more thoughtful and sensitive trial designs, drives more informative and meaningful outcomes from clinical studies, and builds trust between the public, government, and industry stakeholders. This engagement is especially important in the case of rare diseases, where affected individuals and their families face many difficulties getting information, treatment, and support. Dyne Therapeutics is developing therapeutics for people with genetically-driven muscle diseases. During the development of potential treatments for Duchenne muscular dystrophy (DMD) and myotonic dystrophy type 1 (DM1), Dyne sought the opinions of individuals living with these diseases to inform its clinical trial design and to decrease the difficulties that participants and families might experience participating in them.

**Methods:**

Dyne engaged individuals and families living with DMD and DM1 as expert partners in its clinical development programs. Dyne convened panels of affected individuals and care partners/parents of individuals living with DMD (n = 8) or DM1 (n = 18). Workshops focused on how affected individuals and their families evaluate and select clinical trials for participation, the importance, quality, and burden associated with individual trial design elements, participation considerations such as site location and the study visit design, patient privacy, the suitability and scope of travel and participant support programs, and the accessibility of content in the informed consent (or assent) forms. Dyne also engaged the DMD Community Advisory Board (CAB) to collect feedback and advice on designing optimal and meaningful clinical trials and measuring relevant outcomes.

**Results:**

The issues most important to individuals living with DM1 and DMD regarding clinical trials were the ability to participate/access to the trial, perceptions of benefit and risk of trials and potential treatments, the flexibility of participation, clear communication from the sponsor, availability of information from trusted sources, and patient enrollment. In response to the patient advisory workshops and CAB feedback, Dyne refined clinical trial inclusion/exclusion criteria and clinic visit design, developed a travel service program to address the burden of clinical trial travel and enable long-distance and cross-border participation, planned for home visits when feasible, and allowed for adequate rest before clinic visit initiation and between assessments. Additionally, Dyne developed and implemented a transparent and consistent communications plan (including age-appropriate content) for trial participants and community members, and assessed and adjusted procedures to provide maximum participant comfort and lower anxiety, particularly with younger participants.

**Conclusions:**

Ongoing communication with the Duchenne CAB and with DMD and DM1 patient advisory committee members allows Dyne to stay current with disease community perspectives and feedback on the needs and preferences of those affected and has provided valuable insights into the participant experience thereby helping Dyne initiate clinical trials that better meet the needs of affected individuals and their families.

**Supplementary Information:**

The online version contains supplementary material available at 10.1186/s40900-023-00535-1.

## Plain Language Summary

**Why is this important?:**

Including the viewpoint of people living with chronic diseases when developing new therapeutics helps address their specific needs and improve their quality of life. This is very important for rare diseases, where individuals and their families face many challenges getting information, treatment, and support.

**What did we do?:**

Dyne Therapeutics, a company focused on developing potential medicines for rare muscle diseases, actively involved individuals living with Duchenne muscular dystrophy (DMD) and myotonic dystrophy type 1 (DM1) in their drug development process. Dyne organized workshops for individuals and families living with DMD and DM1 and participated in community advisory boards to gather input from patient advocacy organization leaders.

**How did this help?:**

Partnering with individuals and families living with DMD and DM1 helped Dyne improve clinical trial design and reduce the difficulties affected individuals and their families face when participating in these trials. This valuable feedback has allowed Dyne to design clinical trials to better address the needs of those living with DMD and DM1.

**Supplementary Information:**

The online version contains supplementary material available at 10.1186/s40900-023-00535-1.

## Background

Engaging people living with chronic diseases in drug development and regulatory processes is an important step for ensuring that new treatments are designed to deliver clinically meaningful benefits and improved quality of life. This engagement is essential in rare diseases where affected individuals and their families face significant challenges accessing information, treatment, and support [1] since low disease prevalence results in a lack of widespread physician and clinical development expertise and limited numbers of clinical trials [2, 3]. In addition, including affected individuals and care partners can help mitigate the inherent challenges of conducting clinical trials for rare diseases, such as small and disparate patient populations, paucity of natural history data, and lack of validated outcome measures [2]. Patient-focused drug development (PFDD), as initially developed through US Food and Drug Administration (FDA) initiatives [4], and patient-centered clinical trial design were conceived to highlight the value of ‘patient voice’ and promote an environment where researchers, industry sponsors, healthcare providers, decision-makers, and policymakers effectively and consistently include this information in their work with affected communities. Engaging individuals living with disease leads to more thoughtful and sensitive trial designs, drives more informative and meaningful outcomes from clinical studies, and builds trust between the public, government, and industry stakeholders.

### Engagement initiatives

Many engagement initiatives and frameworks exist at the level of government and non-profit funding agencies [e.g., the Strategy for Patient-Oriented Research (SPOR) in Canada [5], Centre for Engagement and Dissemination in the UK [6], and the Patient-Centered Outcomes Research Institute (PCORI) in the US] [7], as public–private partnerships such as the Clinical Trials Transformation Initiative (CTTI) [8] and the Patients Active in Research and Dialogues for an Improved Generation of Medicines (Paradigm) [9], among regulatory agencies [e.g., the FDA PFDD initiatives [4, 10, 11], European Medicines Agency (EMA)] [12], and from payers [e.g., Centers for Medicare & Medicaid Services, (CMS)] [13]. These programs focus on creating a comprehensive, shared picture of disease burden and identifying clinically meaningful outcomes to benefit those affected. They also offer frameworks and resources that measure treatment benefits and facilitate communication and collaboration between affected communities, healthcare providers, drug developers, and regulators throughout the drug development process. There is a continuing evolution of efforts to understand multi-stakeholder needs better and drive best practices for engagement activities in drug development [14–16].

### Benefits of early-stage engagement

Feedback from affected communities at the early stages of clinical development programs can optimize clinical trial feasibility and utility through participant-centered attention to study procedures such as clinic visit design and frequency, review of informed consent forms, study protocol complexity, including the use of placebo arms, the accuracy and relevance of clinical endpoints and patient-reported outcomes, the acceptability of the treatments under study, and the design and scope of travel and participant support programs [17]. Equally important, incorporating the patient voice in rare disease drug development raises awareness of the challenges faced by those affected by rare diseases and provides opportunities for engaged stakeholders to advocate for increased research and resources.

Dyne Therapeutics is a clinical-stage company focused on advancing therapeutics for people with genetic muscle diseases. Using the proprietary FORCE™ platform, Dyne is developing modern oligonucleotide therapeutics designed to overcome challenges in muscle tissue delivery. Investigational therapeutics include those for Duchenne muscular dystrophy (DMD) and myotonic dystrophy type 1 (DM1). Dyne is also pursuing potential therapies for facioscapulohumeral dystrophy, and other applications of the FORCE platform are being explored. Dyne engages individuals and families living with these diseases and patient advocacy organization leaders as expert partners in its clinical development programs.

#### Duchenne muscular dystrophy

DMD is the most common childhood-onset form of muscular dystrophy. A mutation in the *DMD* gene located on the X chromosome results in absence or deficient levels of dystrophin, a protein required to strengthen muscle fibers and protect them from injury as muscles contract and relax. DMD affects approximately 1 in 3500 to 1 in 6000 newborn males globally [18, 19]. Clinical symptoms manifest between 3 and 5 years of age, and progressive lower limb muscle weakness results in loss of ambulation in adolescence, while respiratory muscle weakness may require ventilation support beginning in late teens/early adulthood [20]. Cardio-respiratory complications are a leading cause of death in DMD. Respiratory muscle weakness occurs gradually, typically characterized by sleep disturbance and morning headaches, and is often unnoticed until an acute respiratory event occurs. Similarly, signs of cardiac dysfunction become apparent during the later stages of the disease.

#### Myotonic dystrophy type 1 (DM1)

DM1 is a degenerative multi-system neuromuscular disease arising from the expansion of unstable CTG trinucleotide repeats in the *DMPK* gene that encodes a mutated RNA [21]. The mutated RNA sequesters RNA-binding proteins, including splicing factors, into nuclear foci leading to a spliceopathy that drives the clinical manifestations of DM1 [22, 23]. DM1 is the most common form of muscular dystrophy among adults. However, prevalence estimates vary by geographic and ethnic populations, and delayed and missed diagnoses contribute to difficulties in establishing the true prevalence of DM1 [24, 25]. DM1 is estimated to affect from 1:2100 to 1:8000 people worldwide [24, 26]. While there is high inter- and intra-individual variability in the clinical manifestations of DM1 [27], skeletal muscle, cardiac, respiratory, and the central nervous system (CNS) involvement determine functional limitations and survival.

DMD and DM1 are associated with early mortality, and affected individuals and their care partners experience significantly impaired quality of life. [23, 28].

### Dyne’s DMD and DM1 community engagement efforts

During the clinical development of therapeutics targeting the genetic defects in DMD and DM1, Dyne engaged those living with these diseases to ensure that it had a current and comprehensive understanding of disease burden to increase overall study quality and to minimize participant burden. The approaches to engagement initiatives, examples of the recommendations resulting from these initiatives, and the impact on the clinical development plans are discussed.

## Methods

### Planning

**Fig. 1:**
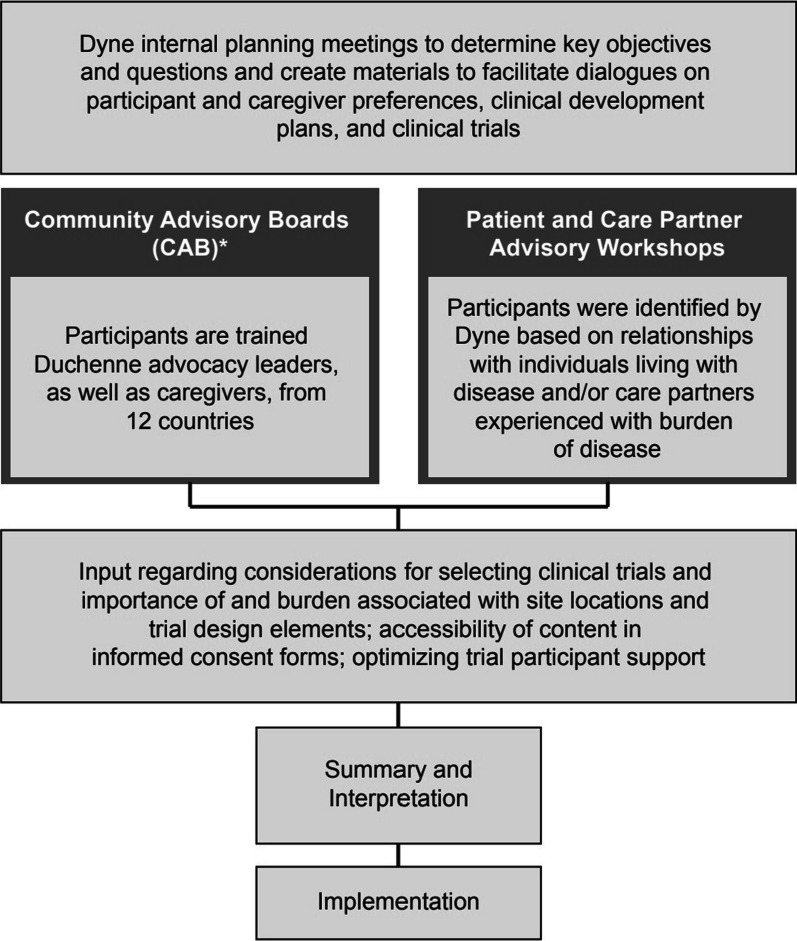
Framework for Dyne planning, execution, and interpretation of engagement activities (* At the time of this analysis, only the Duchenne CAB was providing consultation to Dyne as a DM1 CAB had not yet been formed)

The approach to engagement (Fig. 1) included collaboration with DMD and DM1 communities for guidance on clinical development programs, participant communication, and clinical trial and study visit design for the DELIVER (NCT05524883) and ACHIEVE (NCT05481879) trials. DELIVER consists of a randomized placebo-controlled 24-week period of multiple ascending doses of the investigational therapeutic DYNE-251 for individuals with DMD amenable to skipping exon 51, followed by open-label and long-term extension periods. The ACHIEVE trial has a similar design for assessing DYNE-101 for adults with DM1. The involvement of DMD and DM1 communities follows the Guidance for Reporting Involvement of Patients and the Public (GRIPP2) [29], which was developed to improve the reporting of patient and public involvement in health research (Additional file 1).

Dyne planned workshops with affected individuals and care partners to understand how patients and their families evaluate and select clinical trials for participation, the importance, quality, and burden associated with individual trial design elements, participation considerations such as site location and the study visit design, patient privacy, the suitability and scope of travel and participant support programs, and the accessibility of content in the informed consent (or assent) form (ICF). Some workshop participants were identified in partnership with patient advocacy organizations. Dyne also invited DM1 and DMD community members, some of whom were already serving as advisory partners to the company, to participate in advisory workshops. It also engaged the DMD Community Advisory Board (CAB) to collect feedback and advice on designing optimal and meaningful clinical trials and measuring relevant outcomes.

### Execution

#### Patient advisory workshops

Dyne Therapeutics convened panels of affected individuals and care partners/parents of individuals living with DMD (n = 8) or DM1 (n = 18). For the DMD workshop, four parents of children with DMD from the US and Europe, and four young men from the US with DMD (three who were either in college or post-graduate programs, and one self-employed entrepreneur) participated. Participants in the two DM1 workshops included 5 men and 3 women with DM1 from the US, UK, Germany, France, Belgium and the Netherlands (the youngest in their 30s and the oldest in their 70s; 3 of whom were also parents of adolescents or adults with DM1) and 10 caregivers (parents) of adults with DM1. The majority of DM1 workshop participants had post-graduate degrees, and six worked in healthcare. Participants signed agreements that included confidentiality provisions and were compensated as expert advisors.

The company held pre-workshop planning meetings to refine the key objectives and questions for each session and direct the design of presentations, areas for discussion, and any materials to be reviewed by participants before the advisory workshop. Information reviewed by participants prior to the workshops included workshop objectives, overview of trial designs, and proposed outcome measures. Participants had the opportunity to provide input on agendas and key questions for discussion prior to the meetings. In addition to affected individuals and caregivers, crossfunctional Dyne team members attended the advisory workshops. Facilitated discussions with workshop participants captured feedback and insights on factors summarized in Fig. 1 to assist in the design of the DELIVER and ACHIEVE trials. Workshops were structured to first provide an overview of the Dyne FORCE therapeutic platform followed by overview of the clinical development plan and possible outcome measures for clinical trials. Workshop participants were then asked to consider for each outcome measure the aspect of their disease assessed, any experiences with the assessments, potential burden of functional tests, and in some cases, if participants would consider home wearable devices and video assessments. A specific question regarding outcome measures explored participants experiences with muscle biopsies and how many they would consider undergoing in a 6 month or 1 year period. Participants were questioned on the specifics of placebo and blinding in the clinical trials (e.g., how long they would be willing to be blinded and potentially receiving placebo treatment instead of active study drug). Discussions also focused on preferences for travel to the clinic for study visits. Finally, workshop members provided information on the factors that influenced whether they selected or avoided participation in clinical trials.

#### Community Advisory Board (CAB)

The Duchenne CAB [30] is an independent and autonomous international board of patient representatives (caregivers) from 12 countries established and operated by patient advocates (https://www.duchennedatafoundation.org/project/duchenne-cab/). Members of the Duchenne CAB are selected to represent individuals living with Duchenne across every age and stage of the disease. Patient experts from the World Duchenne Organization member organizations empower the board. The Duchenne CAB provides collective knowledge and expertise with the goal of accelerating research and development, clinical trials, and access to effective treatments for DMD worldwide.

The Duchenne CAB is compensated and provides ongoing consultation to Dyne (meetings are held twice per year) on critical and timely topics related to participant and caregiver preferences, Dyne’s DMD clinical development plans, and the DELIVER clinical trial. CAB members received information on the Dyne clinical development program in order to have discussions and provide feedback on specifics of protocol design, patient education and communication.

A DM1 CAB was unavailable for consultation at the time of Dyne’s development program. However, the development of a DM1 CAB is underway, and Dyne looks forward to engaging with the program in the future to share and receive feedback on its DM1 clinical development program.

### Interpretation

Facilitators and notetakers met after each patient advisory workshop to identify key themes that emerged from the discussions across the workshop topics, highlighting dialogue among participants that illuminated specific concerns or issues within the three main workshop topics (Table 1): trial protocol elements, study visit design, and critical considerations for selecting a clinical trial.

**Table 1:** Feedback on key considerations for trial selection, protocol elements, and study visits

Topic	Top-line discussions
Considerations for enrollment in clinical trials	• The DM1 and DMD communities are willing to participate in trials because of the significant need for therapies
	Advisory participants were supportive of efforts to reduce the considerable travel burden for participants and families:
	• The proximity of the trial site to the home is attractive; car and train travel are significantly preferred over air travel
	• A low frequency of clinic visits/clinic site travel is preferred
	Advisory participants were supportive of the following:
	• A shorter placebo duration and asymmetrical study design (2:1 or 3:1) are preferable
	• Input and support from medical care providers and other families living with disease to help make trial participation decisions
	• Patient advocacy organization communication to create visibility and access to trial information is important to support decision making
	• Access to thorough trial inclusion/exclusion criteria to help make initial eligibility determinations and trial assessments easier
	Trial participation decisions were positively impacted by the following:
	• An understanding of the investigational medicines’ mechanisms of action
	• An understanding of relevant, non-clinical, translational data
	• A clear explanation of the study design and outcomes
	• The opportunity to receive the active drug in an open-label extension of the study
Clinical trial protocol elements (DMD advisory workshop participants and duchenne CAB feedback)	• Care providers primarily valued functional assessments that measure improvements in the ability to perform an activity; they also prefer video assessments
	Advisory participants:
	• Helped Dyne identify appropriate measures to mitigate anxiety that younger participants may experience with certain clinical assessments including MRI, biopsies, and blood draws
	• Required a clear rationale for the need for biopsies, provided advice on how best to educate on the reasons for such a procedure, and were supportive of providing detailed information to trial participants
	• Were supportive of endpoints related to activities of daily living as illustrative and useful in assessing the impact of an investigational therapeutic
	The CAB:
	• Supported proposed plans to minimize the number of placebo participants as much as possible, recognizing that the decision depends on several factors
	• Endorsed the approaches to define a sequential order of assessments for all outcome measures for consistency and intentions for modifying endpoint and outcome measures
Clinical trial protocol elements (DM1 advisory workshop specific feedback)	Advisory participants helped dyne:
	• Identify the multiple factors (e.g., temperature, activity, sleep) that impact accurate quantification of myotonia
	• Identify specific opportunities for education regarding assessments that could be considered more challenging (e.g., where recovery and healing many be needed)
	• Consider the factors such as travel and time of day that influence disease manifestations (including fatigue level, strength, GI symptoms, and overall performance) in order to optimize the design and interpretation of test results
	• Better understand the challenges of performing functional assessments in the clinic
	Advisory participants:
	• Considered pulmonary testing important
	• Considered muscle needle biopsies important to provide accurate assessment of potential therapeutics
Study visits (DMD and DM advisory workshop participants and duchenne CAB feedback)	Advisory participants recommended that Dyne:
	• Provide a detailed itinerary and schedule to ensure a smooth clinic visit
	• Build flexibility in the timing of assessments to help with individual participant needs
	• Minimize travel burden through financial and organizational support (critically important): have travel and patient support programs that provide fit-for-purpose, long-distance and cross-border travel, meals, housing, insurance, and related support programs
	• Provide age-appropriate communication to engage participants
	• Communicate trial status information through research coordinators

CAB, Community Advisory Board; DM1, myotonic dystrophy type 1; DMD, Duchenne muscular dystrophy

After each meeting with the CAB, the CAB provided comprehensive notes and letters of recommendation to Dyne regarding patient education and communication, the clinical development plan and specifics of protocol design, and regulatory issues, including outstanding questions, topics for further discussion, and areas where the CAB was interested in ongoing collaboration and follow-up.

Results from each activity (CAB and patient advisory workshops) were summarized, reviewed, and discussed by Dyne personnel and integrated into the final design of study protocols, clinic visit schedules, ICFs, and clinical development plans. Post-meeting follow-up with participants included summaries of the workshop proceedings and discussions to ensure that input was accurately reflected. CAB meeting summaries were shared and reviewed at subsequent CAB meetings.

## Results

Top-line discussions from patient advisory workshops and CAB meetings on three main topics: trial protocol elements, study visit design, and criteria used by potential participants to evaluate and select clinical trials are summarized in Table 1.

### Analysis

**Fig. 2:**
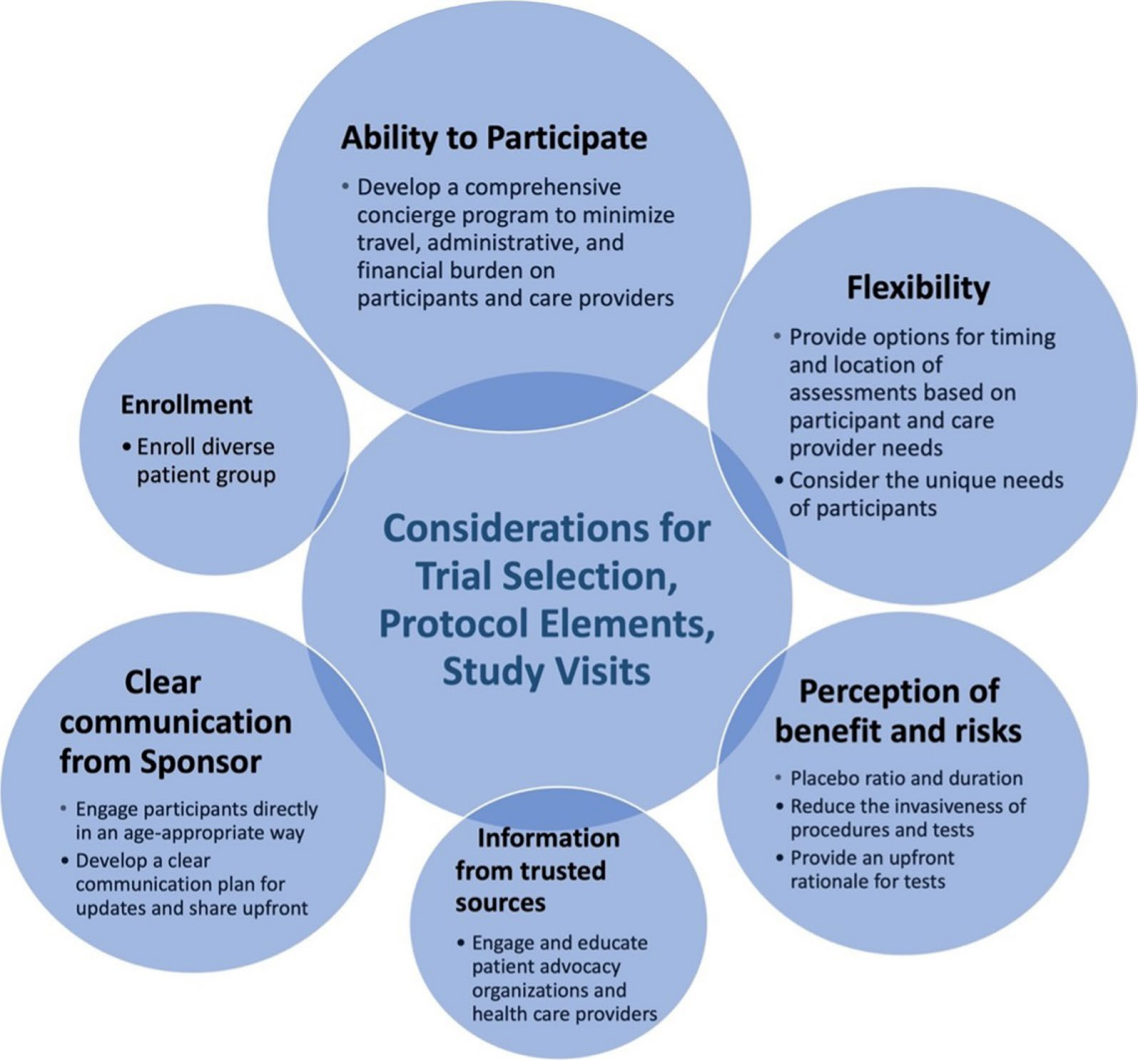
Key themes arising from discussions on the topics of clinical trial selection considerations, protocol elements, and study visits. Size of the circles shows the relative weighting of the feedback received

The Dyne team summarized, reviewed, and grouped CAB and workshop participant feedback according to six common themes that captured the issues of clinical trial participation most important to those living with DM1 and DMD: ability to participate in the trial, perceptions of benefit and risk of trials and potential treatments, the flexibility of participation, clear communication from sponsor, availability of information from trusted sources, and patient enrollment. Figure 2 summarizes these themes and shows the relative weighting of the feedback received within each category.

The DMD-specific and DM1-specific workshops captured similar feedback between the two populations, but some issues were unique to each group regarding protocol elements as summarized in Table 1.

Dyne took specific actions in response to the patient advisory workshop and CAB feedback regarding the clinical development program, clinical trial, and clinic visit design. Examples include:

- refining clinical trial inclusion/exclusion criteria and clinic visit design based on participant feedback related to perceptions of benefits and risks of trial participation;
- developing a travel service program to address the burden of clinical trial travel and enabling long-distance and cross-border participation for increased access to the clinical trial;
- planning for home visits when feasible; ensuring adequate rest before clinic visit initiation and between assessments;
- developing and implementing a transparent and consistent communications plan for trial participants and community members;
- assessing and adjusting procedures to provide maximum participant comfort and lower anxiety, particularly with younger participants, including age-appropriate communication content.

Incorporating feedback from the DMD-specific and DM1-specific workshops was balanced with other factors critical for clinical trial design, including implications of preclinical data dose and dose regimen selection, learnings from previous or ongoing natural history and interventional studies relevant to Dyne’s programs, the impact of statistical analysis planning on the number of participants needed in the trial arms, regulatory guidelines, and reimbursement authority needs.

## Discussion

As experts in living with chronic and progressive diseases, individuals and care partners in DMD and DM1 communities bring unique perspectives to drug development and evaluation of trial designs and outcomes [31, 32]. This is especially important for rare diseases where low prevalence results in a lack of widespread physician and clinical development expertise, and where there may be no disease-altering therapies, there are limited numbers of clinical trials, and there are few validated outcome measures [2, 3]. Affected individuals and families can provide a first-hand understanding to other stakeholders of both the disease burden and the burden associated with trial participation [33]. Consequently, regulatory and payer mandates for patient engagement in drug development are becoming more common. For all stakeholders in the rare disease therapeutic space, engagement has evolved from a “nice to have” to a critical imperative [32]. Biotech and pharmaceutical companies are therefore increasingly urged or directed to include the patient voice in the development of potential therapies to tailor, refine, and ultimately improve the design and execution of interventional studies [31]. However, implementing some recommendations developed by the CAB and patient advisory workshops may be limited by regulatory guidelines on clinical trial design across regions, and other factors such as statistical planning that impact numbers of patients in study arms.

Affected individuals and care partners should be engaged throughout clinical development to provide education on disease and symptom burden, define clinically meaningful benefits, optimize study design and protocols, and improve travel and support programs [17, 34]. Engagement methods may involve unidirectional feedback from patients and care partners via interviews and focus groups, unidirectional and bi-directional consultation with patient and partner advisory boards, and bidirectional collaboration with affected individuals and partner advisers, steering committees, and community-based advocacy groups. The CAB is designed, staffed, and trained to provide country and regional insights on issues and constraints (e.g., regulatory, travel) that impact clinical development programs and clinical trial participation. Dyne’s engagement activities to gather feedback on drug development programs, clinical trial design, and methods to mitigate the significant burden placed on families involved in trials have helped the company refine and hone its understanding of the needs and preferences of affected individuals and their care partners and, ultimately, improve study quality.

While there was overlap in the most important issues for the DMD and DM1 community participants, there were also differences that highlight that neuromuscular diseases are not the same regarding the impact that disease and symptom burden has on affected individuals and their families. For example, the DMD community was especially concerned with mitigating anxiety in younger trial participants, while the DM1 community stressed the challenges for assessing myotonia and the need for recovery time after challenging assessments. Thse differences highlight the fact that caregivers offer unique perspectives regarding the risks and benefits of clinical trial participation and the impact of protocol elements and study visit design, and pediatric caregiver preferences differ from those of adult caregivers. It is necessary, therefore, to design and implement engagement processes that will lead to study protocol and clinical trial design recommendations that are customized and tailored to the disease and community involved in the study. Doing so will also enhance participant and public trust in drug development.

Overall, clinical trial participation places a considerable burden on individuals and families already struggling with life-altering diseases. The results from Dyne’s engagement activities with affected individuals and care partners highlighted key themes of access and affordability, flexibility, communication with sponsors and information from trusted sources, and perception of potential treatment benefits and risks. These themes are similar to other analyses of the burdens of clinical trial participation and issues that individuals consider most important about trial design and participation [35–38]. However, these previous studies did not focus on individuals with rare muscle diseases. Dyne made responsive and targeted changes to implement the feedback from the DMD and DM1 communities to decrease the burden of clinical trial participation by addressing issues related to study visit design, outcome measures, and communication and information-sharing pathways. There is the potential for bias regarding the feedback garnered from CABs and patient workshops since participants in these activities are those within the community who are already actively engaged with patient advocacy organizations and the pharmaceutical industry, and tend to have received secondary education, sometimes in the healthcare arena. However, the broad range of experiences that the participants brought, from caregivers for children and adults living with disease (including some caregivers who are also affected) to those living with disease themselves, provided Dyne with the confidence that their voices accurately reflect the needs of the broader DMD and DM1 communities.

Dyne conducts ongoing communication with the Duchenne CAB and with DMD and DM1 patient advisors to stay current with disease community perspectives and feedback on the needs and preferences of those affected by neuromuscular disease. These engagement activities provide valuable insights into the participant experience and have helped Dyne initiate clinical trials that better meet the needs of affected community members.

### Supplementary Information

**Additional file 1.** GRIPP2 Short Form Checklist.

## Data Availability

Not applicaple.

## Abbreviations

CAB: Community Advisory Board
DMD: Duchenne muscular dystrophy
DM1: Myotonic dystrophy type 1
PFDD: Patient-focused drug development
