# Supplementary material for: Patient engagement in clinical trial design for rare neuromuscular disorders: impact on the DELIVER and ACHIEVE clinical trials
Source: Res Involv Engagem. 2024 Jan 2;10:1. doi: 10.1186/s40900-023-00535-1 (PMC10759564; doi:10.1186/s40900-023-00535-1)
Supplement: Supplementary file 1 — Additional file 1. GRIPP2 Short Form Checklist. [file 40900_2023_535_MOESM1_ESM.docx]

**Additional File 1: GRIPP2 Short Form Checklist**

| **Section and topic** | **Item** | **Reported on page No** |
| --- | --- | --- |
| 1. Aim | Report the aim of PPI in the study | 5-6; 8 |
| 2. Methods | Provide a clear description of the methods used for PPI in the study | 9-12 |
| 3. Study results | Outcomes – report the results of PPI in the study, including both positive and negative outcomes | 13-17 |
| 4. Discussion and conclusions | Outcomes – comment on the extent to which PPI influenced the study overall. Describe positive and negative effects | 18-20 |
| 5. Reflections/critical perspective | Comment critically on the study, reflecting on the things that went well and those that did not, so others can learn from this experience | 17-20 |
